# Identification of *Toxoplasma Gondii* Tyrosine Hydroxylase (TH) Activity and Molecular Immunoprotection against Toxoplasmosis

**DOI:** 10.3390/vaccines8020158

**Published:** 2020-04-01

**Authors:** Zhenchao Zhang, Yuhua Li, Haoran Li, Xiaoxiao Song, Zhongshan Ma, Haoran Lu, Shuyue Liu, Yi Zhao, Mengyao Tan, Shuai Wang, Xiangrui Li

**Affiliations:** 1 Xinxiang Key Laboratory of Pathogenic Biology, School of Basic Medical Sciences, Xinxiang Medical University, Xinxiang 453003, China; 161020@xxmu.edu.cn (Z.Z.); 192028@xxmu.edu.cn (Y.L.); harryli1211@163.com (H.L.); songxiaoxiao712@163.com (X.S.); mazhongshan0318@163.com (Z.M.); 18236352133@163.com (H.L.); lsy18338981571@163.com (S.L.); zy15236286017@163.com (Y.Z.); tmy15537700657@163.com (M.T.); 2 MOE Joint International Research Laboratory of Animal Health and Food Safety, College of Veterinary Medicine, Nanjing Agricultural University, Nanjing 210095, China

**Keywords:** *T. gondii*, tyrosine hydroxylase, catalytic activity, animal challenge, protective immunity

## Abstract

The neurotropic parasite *Toxoplasma gondii* (*T. gondii*) infection can change the behavior of rodents and cause neuropsychological symptoms in humans, which may be related to the change in neurotransmitter dopamine in the host brain caused by *T. gondii* infection. *T. gondii* tyrosine hydroxylase (TgTH) is an important factor in increasing the neurotransmitter dopamine in the host brain. In this study, the enzyme activity of TgTH catalytic substrate for dopamine production and the molecular characteristics of TgTH were identified. In order to amplify the open reading frame (ORF), the designing of the specific primers for polymerase chain reaction (PCR) was on the basis of the TgTH sequence (GenBank Accession No. EU481510.1), which was inserted into pET-32a (+) for the expression of recombined TgTH (rTgTH). The sequence analysis indicated that the gene of TgTH directed the encoding of a 62.4-kDa protein consisting of 565 amino acid residues, which was predicted to have a high antigen index. The enzyme activity test showed that rTgTH and the soluble proteins extracted separately from *T. gondii* RH strain and PRU strain could catalyze the substrate to produce dopamine in a dose-dependent manner, and the optimum catalytic temperature was 37 °C. The result of the Western Blotting assay revealed that the rTgTH and the native TgTH extracted from somatic of *T. gondii* RH tachyzoite were successfully detected by the sera of mice infected with *T. gondii* and the rat serum after rTgTH immune, respectively. Immunofluorescence analysis using antibody against rTgTH demonstrated that the protein was expressed and located on the surface of *T. gondii* RH tachyzoite. Freund’s adjuvant was used to emulsify the rTgTH, which was subsequently applied to BALB/c mouse immune thrice on week 0, week 2, and week 4, respectively. The result of the animal challenge experiments showed an integral increase in IgG, IgG2a, IgG1, and IFN-γ, IL-4, and IL17 were as well significantly increased, and that the rTgTH vaccinated animals apparently had a prolonged survival time (14.30 ± 2.41) after infection with the RH strain of *T. gondii* compared with that of the non-vaccinated control animals, which died within 11 days. Additionally, in the rTgTH vaccination group, the number of brain cysts (1275 ± 224) significantly decreased (*p* < 0.05) compared to the blank control group (2375 ± 883), and the size of the brain cysts in the animals immunized with rTgTH vaccination was remarkably smaller than that of the control mice. All the findings prove that TgTH played an important role in increasing the neurotransmitter dopamine in the host brain and could be used as a vaccine candidate antigen to mediate cell-mediated and humoral immunity.

## 1. Introduction

*T. gondii*, an obligate intracellular apicomplexan parasite, is the causative agent of the anthropozoonotic disease called toxoplasmosis, which can be widely parasitic in a broad range of warm-blooded vertebrates. Toxoplasmosis seriously threatens the health of human and livestock, and it is spread by animals and distributed all over the world [1]. Approximately 30% of the world’s population has been infected with *T. gondii* [2]. Symptoms may be inapparent and are accompanied by the generation of cysts in some hosts infected with less-virulent *T. gondii* strains, but the host infected by virulent strains will suffer from acute toxoplasmosis and may even die. In addition, the infection can cause high rates of morbidity and mortality for some patients and can result in such patients developing AIDS [3]. Additionally, the infection of toxoplasma leads to abortion, stillbirth, as well as death of neonates, causing enormous economic losses [4,5]. Infected livestock are the main route of transmission to humans [6].

The drugs for controlling acute infection of *T. gondii*, such as pyrimethamine, sulphadiazine, and atovaquone, could not eliminate chronic infection [7]. Moreover, the drug resistance of *T. gondii* has appeared and drug residue in animal-derived foods has occurred more and more extensively [8,9,10]. Therefore, it is crucial that we develop new and effective vaccines in humans and animals with *T. gondii* infections. The emergence of safe and effective vaccines will greatly reduce the economic loss of stock-raising industry [8].

Recent studies have suggested that the infection with *T. gondii* was a risk factor for developing psychiatric disorders, such as depression and schizophrenia, as well as human personality and behavior changes and risk of suicide [11,12]. The rodents chronically infected with *T. gondii*, which are a key intermediate host for this parasite, exhibit a distinct repertoire of specific behavioral changes, including a loss of aversion to cat odors [13,14]. The exact behavior regulation mechanism is unknown, but *T. gondii* stimulation of host dopamine pathways in the brain has been suggested as a cause [15,16,17]. Some studies showed that chronic infection of *T. gondii* resulted in 14% increase of dopamine level in the whole brain of mice [18]. Moreover, *T. gondii* infection has been shown to increase the amount of dopamine in the striatum, another region of dense dopamine neurotransmission, of mice, with a 38% greater level in infected mice relative to uninfected mice [17]. Dopamine receptor antagonists, such as haloperidol and GBR 12909, can block the behavior changes of infected mice, and haloperidol also has the activity of anti *T. gondii* [17].

Tyrosine hydroxylase (TH), the rate-limiting enzyme for dopamine synthesis, was also found in intracellular tissue cysts in brain tissue with antibodies specific for the parasite-encoded tyrosine hydroxylase [16]. It was found that *T. gondii* infection did not significantly increase the level of TH mRNA and protein in host cells, nor the phosphorylation of TH [19]. Nevertheless, two genes encoding aromatic amino acid hydroxylases, as TH, were contained in the *T. gondii* genome, which was the rate-limiting enzyme with dopamine synthesis in metazoans by converting tyrosine into the dopamine precursor 3,4 dihydroxyphenylalanine [20]. Immunofluorescence indicated that the host produced dopa decarboxylase and TgTH encoded by *T. gondii* co-located in parasitophorous vacuole. The import of host dopa decarboxylase into the parasite’s compartment without altering the amount of host dopamine biosynthetic enzymes or the phosphorylation status of TH may be a mechanism for the specific regulation of dopamine synthesis in host cells without the detrimental effects of unpackaged cytosolic dopamine [19]. In addition, the strike out of the TgTH genes led to a decrease in *T. gondii* infection, a reduction in oocyst production, and lower rates of sporulation in the cat [21]. Therefore, TgTH not only played an important role in changing the synthesis of dopamine in host cells, but also could be used as a candidate vaccine antigen or drug target against *T. gondii* infection.

In this study, we aim to explore the ability of TgTH catalytic substrate to produce dopamine and identify the immunoprotective properties of TgTH.

## 2. Materials and Methods

### 2.1. Ethical Statements

The experiments in this study were conducted following the guidelines of the Animal Ethics Committee, Xinxiang Medical University, Henan, China (Reference No. 2015016). With the aim of alleviating the distress and pain of experimental animals as much as possible, all efforts were paid. In this research, we regularly observed infected mice, and euthanasia was used at humane endpoints, when the mice appeared to be curled up, fluffy, dirty, and lusterless fur, and unresponsive to external stimuli, for example, they could not get up quickly after being pushed down and lying on their side. Generally, euthanasia was conducted by placing the animals in a closed space and exposing them to 60%–70% CO_2_ for five minutes. Occasionally, cervical dislocation was also used for confirmation of valid euthanasia.

### 2.2. Mice and Parasites

Six-week-old female BALB/c mice were purchased from Beijing Vital River Laboratory Animal Technology Co., Ltd. (Beijing, China) and kept in specific-pathogen-free (SPF) conditions.

The strains of *T. gondii* RH (type I) and PRU (type II) were provided by the Department of Human Parasitology of Xinxiang Medical University in Henan, China. The RH tachyzoites were harvested from human foreskin fibroblast (HFF) cells maintained in our laboratory, and HFF cells were cultured in DMEM supplemented with 2% FBS. The *T. gondii* PRU strain was kept by the passage of cysts in BALB/c mice.

### 2.3. Preparation of Soluble Tachyzoite and Bradyzoite Antigens

As described previously, soluble tachyzoite and bradyzoite antigens was prepared from tachyzoites and bradyzoites of *T. gondii* [22]. Briefly, tachyzoite and bradyzoite cells were repeatedly frozen and thawed three times under the temperature of −20 and 4 °C, respectively. Afterwards, the lysate was dispersed by ultrasound with the speed of 60 W/s on ice and separated by centrifuge at 14,000× *g* for half an hour under the temperature of 4 °C. The supernatant was collected and filtered for sterilization and the final soluble tachyzoite and bradyzoite antigens were divided into equal volume and stored at −70 °C for standby. The concentration of soluble antigens was determined by Bradford method with the standard of bovine serum albumin (BSA).

### 2.4. Total RNA Extraction of T. Gondii

The extraction of total RNA of *T. gondii* RH strain trophozoites was conducted by means of E.Z.N.A.^TM^ Total RNA Kit I (OMEGA, Zhengzhou, China) in strict accordance with the manufacturer’s instructions. Water treated by diethyl pyrocarbonate (DEPC) and added with ribonuclease inhibitor (TaKaRa, Dalian, China) was used to resuspend the RNA samples and RNase-free DNase I (TaKaRa, Dalian, China) was used to treat the samples before conducting reverse transcription in order to avoid the contamination from genomic DNA. The OD260 was measured to quantify the RNA samples and the ratio of OD260/OD280 was used to determine the quality of which. The value of OD260/OD280 ranged from 1.9 to 2.0 was considered acceptable.

### 2.5. The Amplification of the ORF of TgTH

The cDNA was amplified by performing RT-polymerase chain reaction (PCR). The primers anchoring protective bases (italic) and restriction enzymes (underlined) (*Eco*R I anchored forward primer, 5′-*CCG*GAATTCATGTCTCTATCCACCGCGCTG-3′; *Hin*d III anchored reverse primer, 5′-*CCC*AAGCTTCTAGATCTTGAGGGAGACAGGAGGC-3′) were used in PCR to amplify the entire ORF of TgTH (GenBank accession no. EU481510.1) from trophozoite cDNA. The ORF of TgTH amplified by PCR was cloned into pMD19-T vector (TaKaRa, Dalian, China) and transformed into *E. coli* (DH5a) competent cells (Yi Fei Xue Biotechnology, Nanjing, China). The recombinant pMD19-T-TgTH clone was amplified in PCR and digested for identification and 3 positive clones that were sequenced for further confirmation. The online tool BLAST (http://www.ncbi.nlm.nih.gov/BLAST/) was used to analyze the homologous sequences of the insertion in the recombined plasmid using GenBank database.

### 2.6. Analyzing Sequences

The similarity between sequences was evaluated by BLASTX and BLASTP (https://blast.ncbi.nlm.nih.gov/Blast.cgi). CLUSTALW1.8 was used to align the sequences of adhesion proteins. The following online tools were applied to the prediction of motifs, secondary structure and signal peptides: Motifscan (http://www.myhits.isb-sib.ch/cgibin/motif_scan), PSIpred (http://www.bioinf4.cs.ucl.ac.uk:3000/psipred/), GPI Modification Site Prediction (http://mendel.imp.ac.at/sat/gpi/gpi_server.html), TMHMM (http://www.cbs.dtu.dk/services/TMHMM/), and SignalP (http://www.cbs.dtu.dk/services/SignalP/).

### 2.7. Expressing and Purifying the Proteins TgTH and pET-32a

*Eco*R I and *Hin*d III was used to digest the recombined plasmid pMD19-T-TgTH after identification. The plasmid pET-32a-TgTH was generated by cloning the purified TgTH fragments into the expressing vector of pET-32a (+) (Novagen, Merck, Germany) after digestion. Sequencing was conducted for the confirmation of TgTH being inserted properly in the right reading frame of the recombined plasmid. After transferring the correct recombined plasmid pET-32a-TgTH into *E. coli* BL21 (DE3) in competence, 0.8 mM Isopropyl-b-D-thiogalactopyranoside (IPTG; Sigma-Aldrich, St. Louis, MO, USA) was added into the culture when reaching the OD600 of 0.6 under the temperature of 37 °C to induce the expression of recombined protein. After five hours of incubation with IPTG under the temperature of 37 °C, the bacteria were centrifuged for harvest. The pellets were treated by lysozyme with the concentration of 10 mg/mL (Sigma-Aldrich, St. Louis, MO, USA) and then sonicated for protein extraction. The protein extract was isolated using 12% (*w*/*v*) sodium dodecyl sulfate polyacrylamide gel electrophoresis (SDS-PAGE).

An Ni2^+^-nitrilotriacetic acid (Ni-NTA) column (GE Healthcare, GE Healthcare Life Sciences, Marlborough, MA, USA) was used to purify the protein of recombined TgTH strictly based on the manufacturer’s instructions, and 12% SDS-PAGE was used to determine the protein purity. The Bradford procedure [23] was applied to the determination of TgTH protein concentration with the standard of BSA. The obtained protein was kept under the temperature of −20 °C for further usage.

Similarly, *E. coli* BL21 after the transformation of pET-32a (+) plasmid was induced to obtain the pET-32a protein with fusion of 6 histidines and Trx•Tag^TM^ thioredoxin protein containing 109 amino acid residues.

### 2.8. Enzyme Activity Test

In strict accordance with the manufacturer’s instructions, an enzyme activity test kit (Haling Biologlcal Technology Co., Ltd., Shang Hai, China) was used to analyze the ability of the rTgTH and soluble proteins extracted separately from *T. gondii* RH strain and PRU strain catalytic substrate to produce dopamine. The set concentration, temperature gradient, and all tests were completed in triplicate.

### 2.9. Antisera against Recombined TgTH and T. gondii

SD rats purchased from Beijing Vital River Laboratory Animal Technology Co., Ltd. (Beijing, China) were subjected to subcutaneous injection into multiple sites with the mixture of Freund’s complete adjuvant and 0.3 mg pure recombined TgTH protein (ratio 1:1) for generating polyclonal antibody. Fourteen days later, the rats were subjected to booster injection once containing the mixture of Freund’s incomplete adjuvant and the same antigen (ratio 1:1) and re-booster thrice with the interval of 7 days. After the entire immune procedure, sera were obtained and conserved for further use. Serum for the negative control was obtained before injection [24].

Mice subjected to the experimental infection of *T. gondii* PRU strain were used to collect antiserum against *T. gondii* (mice antisera) ten days post-infection.

### 2.10. Analyses of Natural and Recombined TgTH by Immunoblot

SDS-PAGE was used to separate the samples that contained recombined TgTH and soluble tachyzoite antigens of *T. gondii*, respectively, and nitrocellulose membrane (Millipore, Shanghai, China) was used in protein hybridization after transferring the proteins in gel. The membranes were subjected to the block of Tris-HCl- Tween buffer solution (TBST), which contained Tween 20, Tris-buffer saline, and 5% (*w*/*v*) skim milk powder, and were then incubated with mouse antiserum (1:100) and rat antiserum (1:200) separately, which were used as the primary antibodies for one hour under the temperature of 37 °C. After that, TBST was used to wash the membranes thrice, and horseradish peroxidase (HRP)-conjugated goat anti-mouse IgG and HRP-conjugated goat anti-rat IgG (Sigma, Shanghai, China) were applied, respectively, to the incubation of the membranes for 1 h under the temperature of 37 °C. In the end, a 3,3′-diaminobenzidine tetrahydrochloride (DAB) kit (Boster Bio-technology, Wuhan, China) was used to detect the bands strictly based on the manufacturer’s instructions.

### 2.11. Expression and Location of TgTH in the Tachyzoite of T. Gondii by Immunofluorescence

Harvested *T. gondii* tachyzoite cells were washed three times with PBS (pH 7.2) and were smeared on a glass slide treated with poly-L-lysine for 15 min. Then, the tachyzoites were subjected to fixation using PBS containing 4% paraformaldehyde for ten minutes under room temperature, permeabilization using PBS containing 1% TritonX-100 for ten minutes, three times of washing by PBS and blockage using PBST containing 4% (*w*/*v*) BSA for one hour under the temperature of 37 °C. After the slides were washed thrice using PBS, rat antiserum against TgTH (diluting ratio 1:100) and blank rat serum were added severally to incubate the slides overnight under the temperature of 4 °C. The slides were washed thrice by PBS, treated by goat anti-rat IgG antibody (Beyotime, Shanghai, China) with Cyanine 3 (Cy3) labeling (dilution ratio 1:1000), and kept in a dark place for forty minutes. After three washes in PBS, 4′,6-diamidino-2-phenylindole (DAPI, Beyotime, Shanghai, China) was to stain the nucleus for 15 min in darkness. After three washes in PBS, fluorescent mounting medium (Beyotime, Shanghai, China) was added and cells were examined by fluorescence microscope (Nikon, Beijing, China).

### 2.12. Immunization and Challenge Infection

Totally, 80 BALB/c mice aged 6 weeks old were classified at random into 4 groups (20/group) and subjected to subcutaneous injection containing 100 μg of recombined TgTH protein and Freund adjuvant (1:1), pET-32a protein mixed with Freund adjuvant (1:1), or the same volume of Freund adjuvant alone separately. The rest of the mice were used as the blank control, which received no inoculation. The animals of all groups were subjected to vaccination thrice on week 0 (Freund’s complete adjuvant was used), week 2 (Freund’s incomplete adjuvant was used), and week 4 (Freund’s incomplete adjuvant was used) separately. Ten days after the last vaccination, the twenty mice of each experiment and control groups were challenged, in which ten were challenged intraperitoneally 1 × 10^3^ tachyzoites of *T. gondii* RH strain, while the other ten animals were challenged intragastrically with 10 cysts of PRU *T. gondii*. The record of survival rate and duration was taken day-by-day. Animals showing symptoms were subjected to euthanasia by CO_2_.

The animals subjected to challenging by RH *T. gondii* were under observation and the record of surviving duration was kept. After 30 days, the survival rate of animals challenged with the PRU *T. gondii* was observed, and the brains of these mice were dislodged and grinded in one milliliter of PBS. A total of 10 μL of brain mixture from each mouse was used for counting cyst quantity with thrice. The size of the brain cysts (*n* = 10) in each group was measured with a microscope and presented by the diameter. The decreased ratio of the number and size were calculated as follows: the number of cysts from the blank control mice–vaccinated mice/the blank control mice×100%, and the size of cysts from the blank control mice–vaccinated mice/the blank control mice×100%, respectively.

### 2.13. The Use of Enzymelinked Immunosorbent Assay (ELISA) for Determining Antibody Levels inSsera

The collection of mouse blood samples in each group (*n* = 5) was conducted on week 0, week 2, week 4, and week 6, and the obtained serum samples were conserved at −20 °C in order to further evaluate antibodies and measure cytokines. With the aim of detecting IgG isotypes and specific anti-TgTH antibodies, indirect ELISA was conducted on the basis of a previous literature with a few details modified [25]. In brief, microtiter plates (Costar, New York, NY, USA) were coated with recombinant TgTH in carbonate buffer (2.5 μg/mL, 100 μL/well) with the PH value of 9.6 overnight under the temperature of 4 °C and blocked with 4% BSA for 2 h at 37 °C. The mouse serum dilution (ratio = 1/10, PBS was used as the diluent) was used to incubate the plates under the temperature of 37 °C for 2 h. After the plates were washed three times by PBST, the plates were treated by second antibodies, which included goat anti-mouse IgG2a, IgG1, IgG, with conjugation with HRP (SouthernBiotech, Birmingham, AL, USA). Finally, the plates were incubated for twenty minutes with the addition of 100 μL of 3,3,5,5-tetramethylbenzidine into each well, and then 100 μL (2 M) of sulfuric acid was added to terminate the reaction. The light absorption at 450 nm was measured by an automatic ELISA reader (Thermo scientific, Waltham, MA, USA) and all tests were completed in triplicate.

### 2.14. Cytokine Determination

The cytokine expressions were determined using the preparation of the serum samples of all the experimental rodents. Ready ELISA kits (Boster, Wuhan, China) were used to measure interferon gamma (IFN-ɣ), interleukin-2 (IL-2), interleukin-4 (IL-4), interleukin-10 (IL-10), and interleukin-17 (IL-17). Recombined IFN- ɣ, IL-17, IL-10, IL-4, IL-2 were used to construct corresponding standard curves with which the cytokines were quantified. The analyses were conducted based on data obtained from 3 individual experiments. 

### 2.15. Analyses of Statistics

The SPSS statistical software (SPSS Inc., Chicago, IL, USA) was utilized to determine the significance of the statistics of Duncan’s multiple range test and one-way analysis of variance (ANOVA), for example, the expression of cytokines and corresponding antibody levels. The survival periods were compared using the Kaplan–Meier method. The tests of differences among groups were conducted, and the threshold value of *p* < 0.05 indicated that the difference was statistically significant.

## 3. Results

### 3.1. Cloning and Sequence Analysis of TgTH

**Figure 1:**
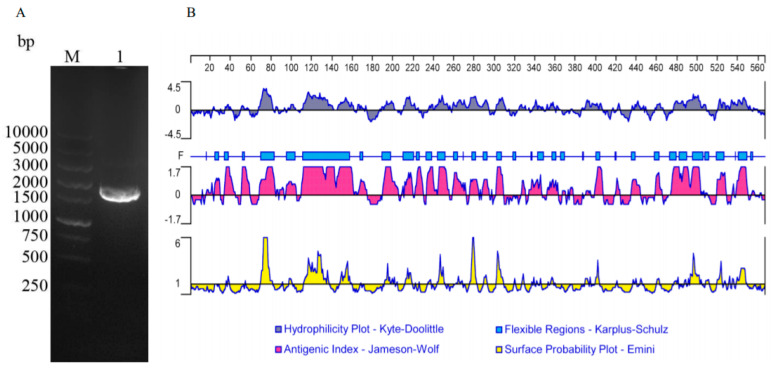
Amplification and bioinformatics analysis of the *gondii* tyrosine hydroxylase (TgTH) gene. (**A**): Agarose gel electrophoresis of the TgTH open reading frame (ORF). (Lane M) DNA molecular weight marker DL 10,000 (ordinate values in bp); (Lane 1) the ORF of TgTH; (**B**): The linear-B cell epitopes of TgTH predicted by DNASTAR in a hydrophilicity plot, flexible regions, antigenic index, and the surface probability rules.

A 1698 bp ORF of TgTH, which encode a protein of 565 amino acids with a molecular mass of 62.40 kDa, was found that started from the ATG initiation codon and ended at the stop codon of TAG by gel electrophoresis (Figure 1A) and sequencing. By analysis with the sequence, it was discovered that the ORF of TgTH contained 60 amino acid residues of strong basicity, 64 amino acid residues of strong acidity, 198 amino acid residues of hydrophobicity, and 154 amino acid residues of polarity with the theoretical pI of 6.76. In comparison to the known sequences of proteins and nucleotides recorded by the National Center for Biotechnology Information (NCBI) database (http://www.blast.ncbi.nlm.nih.gov/blast.cgi/), the identity of the TgTH nucleotide sequence was 99% to *T. gondii* phenylalanine-4-hydroxylase (XM_018779571.1). The TgTH sequence of amino acids exhibited 99% homology compared with the phenylalanine-4-hydroxylase of *T. gondii* (XP_018634670.1) in NCBI. In the deduced protein, two Oglycosylation sites and sixty-four phosphorylation sites could be detected, but no transmembrane domains, GPI anchors, or signal peptides were discovered. As shown in Figure 1B, the protein had six hydrophilicity regions, including 67–160, 186–224, 235–310, 386–405, 476–508 and 520–558, and nine high antigenic index and consecutive regions, including 15–57, 68–173, 189–308, 326–377, 398–406, 434–443, 474–509, 519–527 and 534–556, and most regions of TgTH were flexible regions. It also contained one biopterin-dependent aromatic amino acid hydroxylase region, one domain of phenylalanine 4-monooxygenase, two ACT domains, and three regions of phenylalanine-4-hydroxylase.

### 3.2. Expressing and Purifying Recombined TgTH

**Figure 2:**
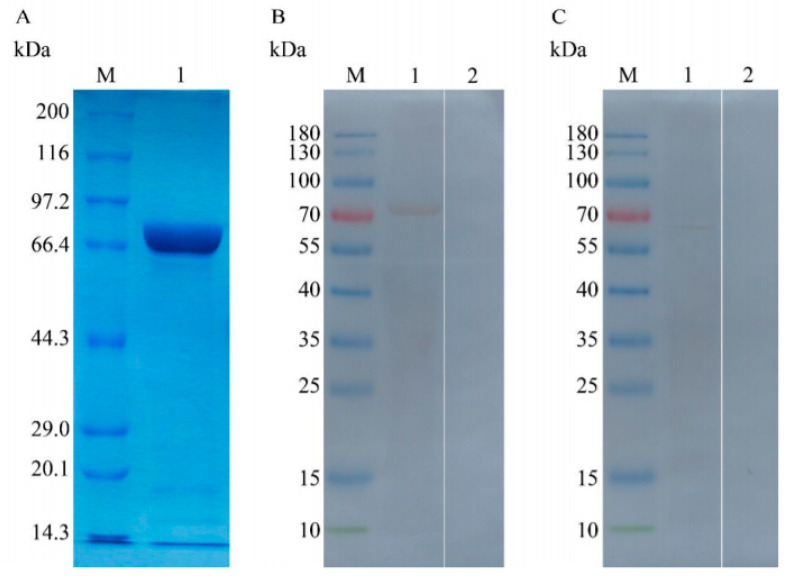
(**A**): Sodium dodecyl sulfate polyacrylamide gel electrophoresis (SDS-PAGE) of the rTgTH protein purified. (Lane M) protein molecular weight marker (ordinate values in kDa); (Lane 1) the rTgTH protein; (**B**): Immunoblot for the rTgTH protein. (Lane M) protein molecular weight marker (ordinate values in kDa); (Lane 1) rTgTH protein probed by serum from mice experimentally challenged with *T. gondii* as the primary antibody; (Lane 2) rTgTH protein probed by the serum of normal mice as the primary antibody; (**C**): Immunoblot for the soluble antigens of *T. gondii* tachychite cells. (Lane M) protein molecular weight marker (ordinate values in kDa); (Lane 1) soluble antigens of *T. gondii* tachychites probed by serum from rat immunized by rTgTH protein. (Lane 2) soluble antigens of *T. gondii* tachychites probed by the serum of normal rats without immunizing as a primary antibody.

The SDS-PAGE result revealed that the supernatant of bacterial sonication extraction exhibited the highest abundance of recombined TgTH, which was subsequently purified from the supernatant using Ni-NTA chromatography and isolated by SDS-PAGE gel as an individual band with the molecular weight of 80.40 kDa (Figure 2A). The molecular mass of the recombined protein accorded with the deduced value of 62.40 kDa after subtracting the fused protein of 18 kDa.

### 3.3. Analysing the Native and Recombined TgTH Using Immunoblot

It was revealed by the immunoblot assay that the recombined protein of TgTH was recognized by serum from mice subjected to artificial *T. gondii* infection instead of serum from healthy mice (Figure 2B). The results of the Western Blot also revealed the recognition of rat anti-TgTH serum to native TgTH protein, which was a band with a molecular mass of around 68 kDa in the somatic extraction of *T. gondii* tachychites (Figure 2C), and a little bit bigger than the deduced protein.

### 3.4. Enzyme Activity Analysis of Dopamine Production

**Figure 3:**
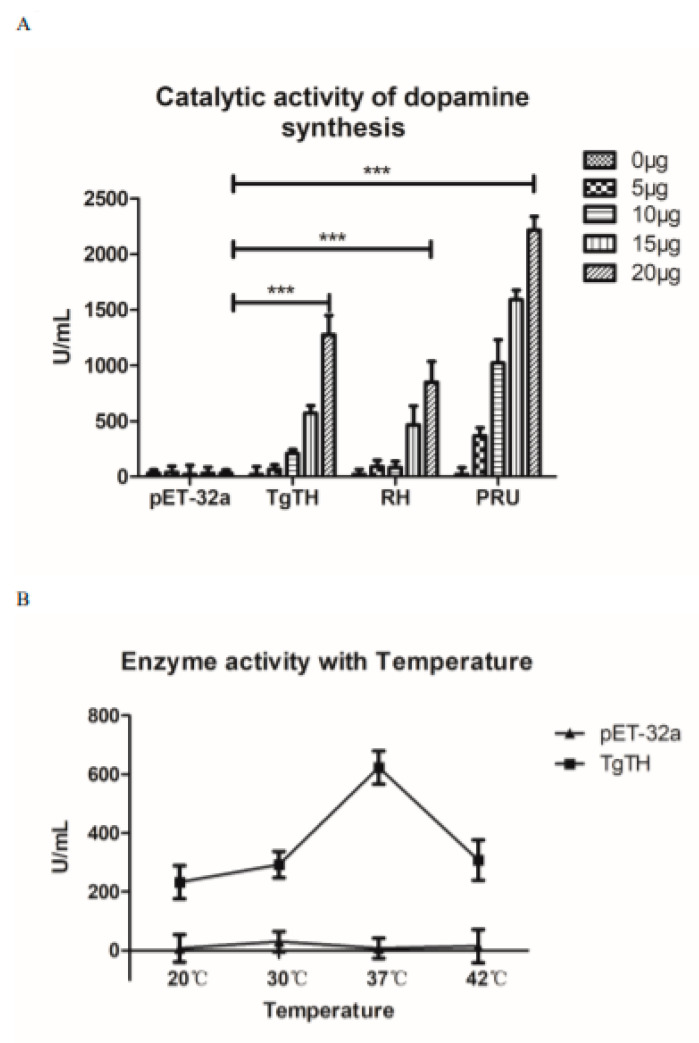
Enzyme activity analysis of dopamine production. (**A**): The rTgTH protein and the soluble proteins from *T. gondii* RH and PRU could catalyze the production of dopamine, and this catalytic activity was dose dependent. Moreover, the soluble proteins extracted from the PRU strain showed the strongest catalytic activity. The result was expressed as mean ± SD. The statistically significant differences (*p* < 0.001) is indicated by (***). (**B**): The catalytic activity of 15 μg TgTH at different temperatures.

In order to explore whether TgTH can catalyze the production of dopamine, the activity of rTgTH and the soluble proteins extracted separately from RH and PRU *T. gondii* were detected in 0 µg, 5 µg, 10 µg, 15 µg, and 20 µg. As show in Figure 3A, compared with the pET-32a protein control group, the rTgTH protein and the soluble proteins from RH and PRU *T. gondii* could catalyze the production of dopamine, and this catalytic activity was dose dependent. In the case of the same amount of protein, the soluble proteins extracted from PRU strain showed the strongest catalytic activity. Moreover, the optimum catalytic temperature for TgTH was 37 °C (Figure 3B).

### 3.5. Expressions and Location of TgTH in Tachychites of T. Gondii

**Figure 4:**
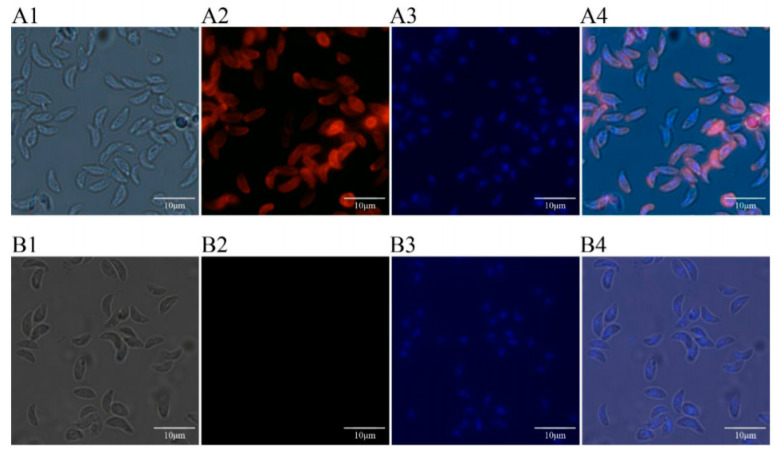
Expression and location of TgTH protein in *T. gondii* tachychites by immunofluorescence assay (×100 magnification). Panel A: *T. gondii* tachychites were probed by the serum of rats immunized by TgTH protein. (**A1**) Differential interference contrast (DIC). (**A2**) Immunofluorescence localization using Cy3. (**A3**) Dyeing nucleus by DAPI. (**A4**) DIC, Cy3, and DAPI combined. Panel B: Negative control, the tachychites were probed by the serum of normal rats without immunizing as the primary antibody. (**B1**) DIC. (**B2**) Cy3. (**B3**) DAPI. (**B4**) Combined.

The expressions and location of TgTH protein was investigated in tachychites (Figure 4) with anti-rTgTH serum. It was revealed that the tachychites were stained by fluorescent material instead of slides in the negative control group and the location of TgTH was principally on the surface of tachychites.

### 3.6. Evaluating the Protective Effect of Inoculation on theE rodents against Challenge of Pathogen

**Figure 5:**
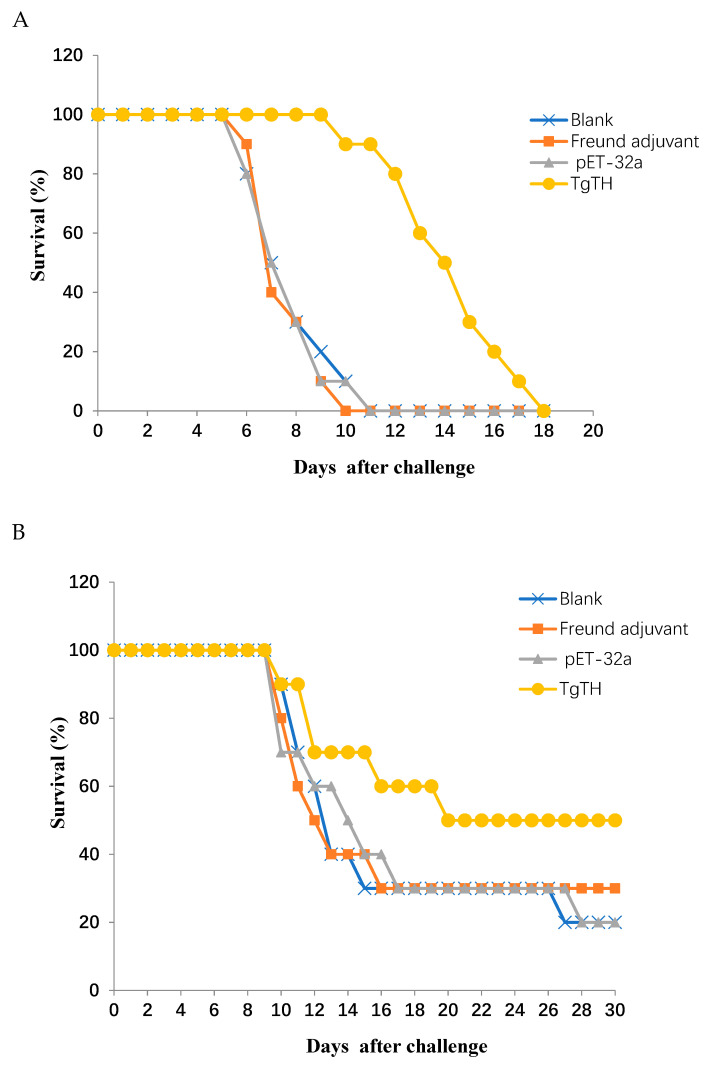
Survival curve of animals after challenging with RH and PRU strain *T. gondii*. The mice were challenged with (**A**) 10^3^ tachyozoite cells of the RH *T. gondii* intraperitoneally and (**B**) 10 cysts of PRU *T. gondii* intragastrically two weeks after the 3rd immunization, respectively.

In order to evaluate TgTH protection on immunized animals against *T. gondii*, the survival and the number of cysts were recorded after the mice were infected by 10^3^ tachyzoites of RH and 10 cysts of PRU *T. gondii*. The survival curves of animals in different groups are shown in Figure 5. The surviving duration of mice immunization with TgTH (14.30 ± 2.41 days, *p* < 0.05) was significantly longer than that of immunization with Freund adjuvant and pET-32a protein (Figure 5A). As shown in Figure 5B, five mice survived in the TgTH group, four weeks after intragastrical infection with 10 cysts of the PRU *T. gondii*. However, these remaining mice in the group of blank, Freund adjuvant, and pET-32a protein were 2, 3, and 2, respectively.

**Figure 6:**
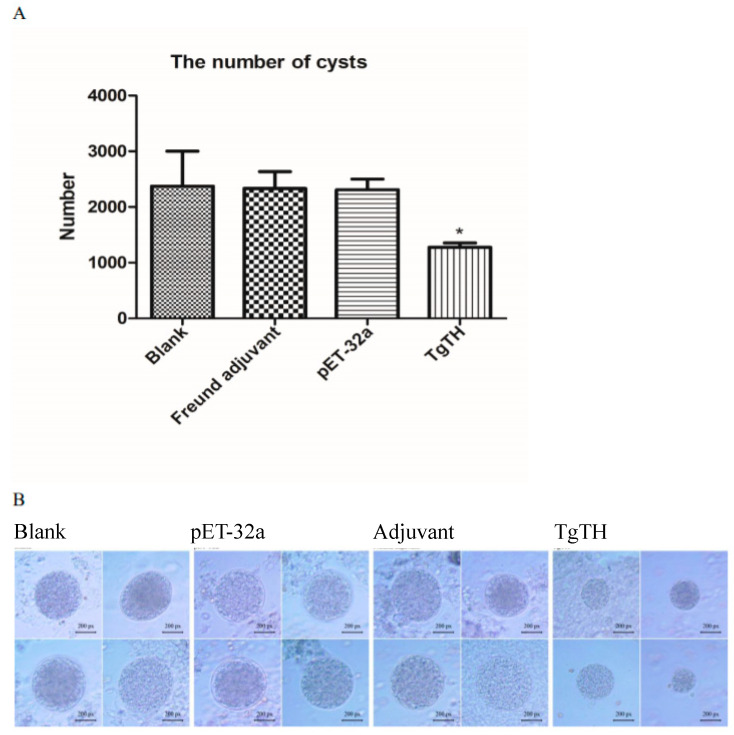
Number and size of brain cysts in the mice after challenging with PRU *T. gondii*. (**A**): The number of cysts in brain was determined and the result was expressed as mean ± SD. Statistically significant differences (*p* < 0.05) is indicated by (*). (**B**): The size of cysts in brain was observed with a microscope (px: pixel).

In the groups of chronic infection, 30 days after oral challenge with 10 cysts of PRU *T. gondii*, the surviving animals were euthanized, and the brain cysts were counted. As shown in Table 1 and Figure 6A, the number of cysts in the brains (1275 ± 224) of the animals in the TgTH group significantly decreased (*p* < 0.05) compared to the blank control group (2375 ± 883). In addition, the size of the brain cysts in the TgTH group was remarkably smaller than the groups of blank, Freund adjuvant, and pET-32a protein (Figure 6B).

### 3.7. Humoral Immunoreactions

**Figure 7:**
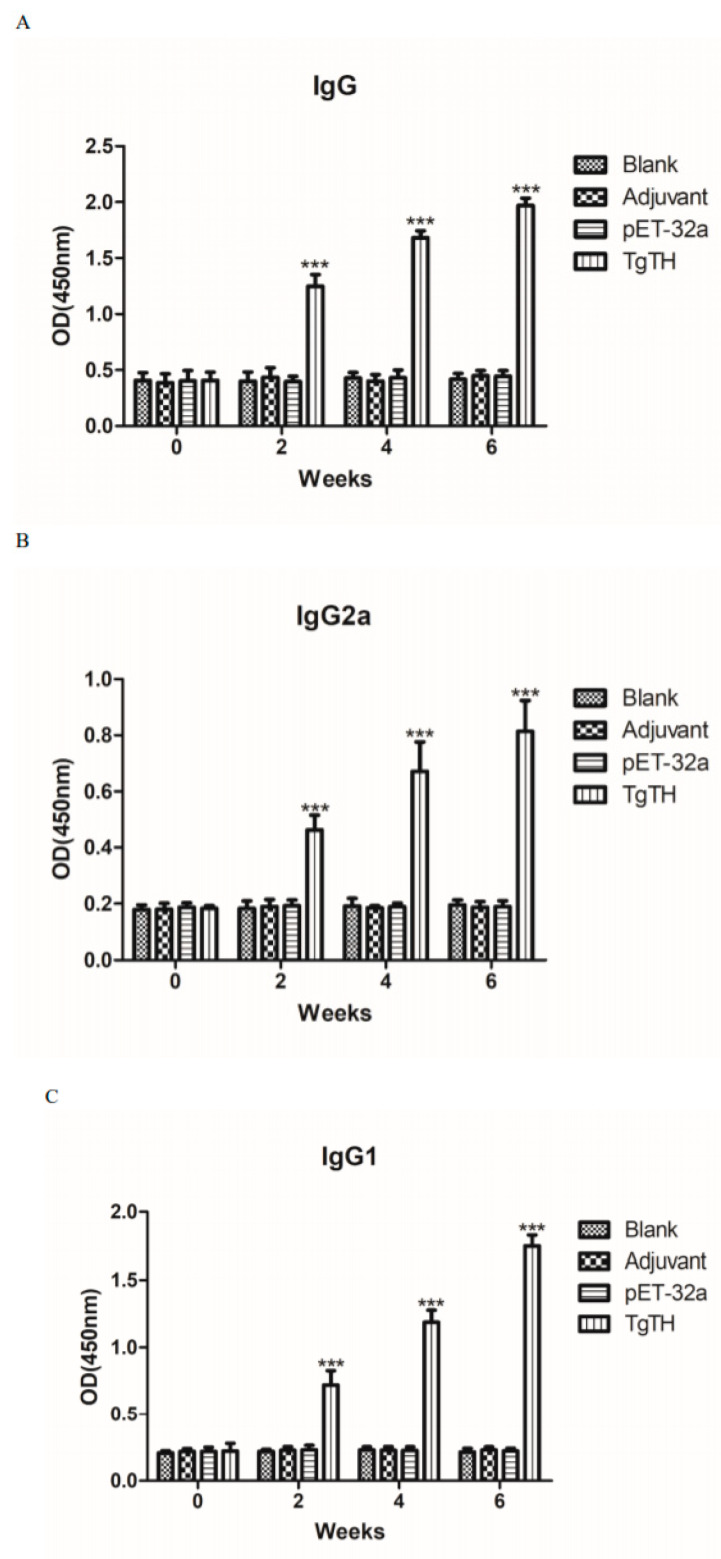
Dynamic humoral immunoreaction of BALB/c mice under the induction of recombined TgTH protein. The BALB/c mice were classified into 4 groups at random and each of which contained 5 mice (*n* = 5). In the three experimental groups, mice received the immunization of Freund adjuvant accompanied with the recombined protein TgTH (1:1), Freund adjuvant accompanied with pET-32a protein (1:1), and Freund adjuvant alone, and the fourth group was used as a blank control. The titers of IgG and the subclass IgG2a and IgG1 were detected on weeks 0, 2, 4, and 6. As for the absorption at 450 nm, the expression pattern was mean ± SD. As for the differences with statistical significance between groups in the identical time point, (***) referred to (*p* < 0.001). (**A**): IgG. (**B**): IgG2a. (**C**): IgG1.

In order to evaluate the varied antibody expressions caused by three sequential vaccines, the distribution pattern of IgG2a and IgG1 isotypes as well as the IgG quantity were examined each time after injection. In comparison to the control group, the IgG quantities in the serum of mice injected with TgTH were significantly higher in statistics (*p* < 0.001). Moreover, the IgG increased when the animals were injected with TgTH continually, and the IgG titers reached the peak at the 3rd immunization. No significant differences were discovered in IgG levels among the control groups (Figure 7A). In comparison to the mice in the control groups, the levels of IgG2a and IgG1 from mice subjected to TgTH injection were among the highest (*p* < 0.001; Figure 7B,C), and in which IgG2a was obviously inferior to IgG1, suggesting the induction of Th2-type cellular immunity by TgTH.

### 3.8. Cytokine Levels in Sera of Immunized Mice

**Figure 8:**
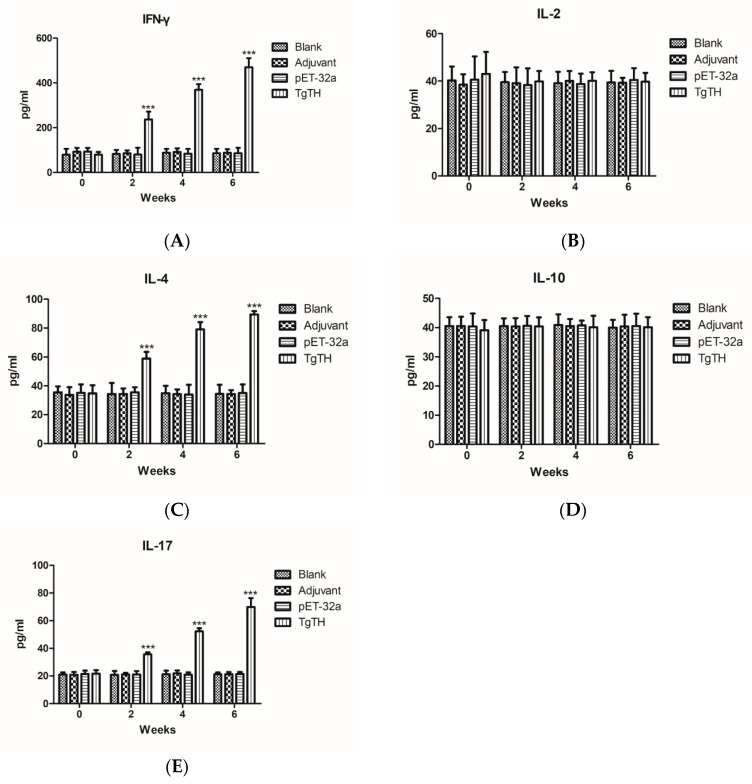
The BALB/c mice were randomly divided into four groups of five mice each (*n* = 5). The animals in each group were immunized with recombinant protein TgTH mixed with Freund adjuvant (1:1), pET-32a protein mixed with Freund adjuvant (1:1), Freund adjuvant alone, and the rest of mice were blank controls. The levels of cytokine were determined by ELISA on 0, 2nd, 4th, and 6th weeks, and the results were expressed as the mean ± SD of concentration (pg/mL). As for the differences with statistical significance between groups in the identical time point, (***) referred to (*p* < 0.001). (**A**): IFN-γ. (**B**): IL-2. (**C**): IL-4. (**D**): IL-10. (**E**): IL-17.

As depicted in Figure 8, the serum samples collected on week 0, 2, 4, and 6 were used to measure the levels of IFN-γ, IL-2, IL-4, IL-10, and IL-17 of the experimental groups. The results indicate that the quantities of IFN-γ, IL-4 and IL-17 (Figure 8A,C,E) of mice injected with TgTH were significantly higher than those of control groups on week 2, 4, and 6 after vaccination (*p* < 0.001), and the levels of IFN-γ, IL-4 and IL-17 were highest throughout the whole experimental after the 3rd immunity. However, the IL-2 and IL-10 levels of the experimental groups were not significantly different from those of the controls (Figure 8B,D).

## 4. Discussion

The protozoan *T. gondii* provides a convincing example of manipulation that selectively alters the host behavior to enhance their transmission [26]. However, the mechanism responsible for these changes remains unclear. Previous studies indicated that anti-dopaminergic drugs could prevent the development of the behavior changes in rodents, which suggested that dopamine regulation altered by the infection of *T. gondii* [27]. As the rate-limiting enzyme in dopamine synthesis, there was no significant increase in the TH expression and phosphorylation of host cells with *T. gondii* chronic infection [19]. The *T. gondii* genome contained two aromatic amino acid hydroxylase genes, which encoded proteins that produced 3,4 dihydroxyphenylalanine (LDOPA) [21]. Moreover, the host produced dopa decarboxylase and TgTH encoded by *T. gondii* co-located in parasitophorous vacuole. Ablation of the TgTH genes resulted in reduced *T. gondii* infection in the cat, lower oocyst yields, and decreased rates of sporulation [21]. Whether TgTH can catalyze the production of dopamine and possess the immunoprotective effect against toxoplasmosis has not been determined.

In the current study, the ORF of TgTH was 1698 bp, which encoded 565 amino acid residues. The molecular weight of TgTH is 62.40 kDa. Based on the analysis result of the protein sequence of TgTH by DNASTAR, TgTH was of intense antigenicity due to outstanding surface probability and antigenic index resulting from the extensively distributed hydrophilic and flexible regions in the protein.

The alignment between TgTH and these sequences in NCBI databases showed that both the nucleotide and protein sequence of TgTH had 99% homology to the nucleotide and protein sequence of phenylalanine-4-hydroxylase of *T. gondii*, respectively. In addition, it was revealed by results of BLASTX and BLASTP that one biopterin-dependent aromatic amino acid hydroxylase region, one domain of phenylalanine 4-monooxygenase, two ACT domains, and three phenylalanine-4-hydroxylase regions existed in TgTH sequence. Whether TgTH possesses the catalytic activity of these enzymes needs further study.

Additionally, sequence analysis showed there were almost no similarities in epitope sequences of amino acid residues between TgTH and the proteins of human homolog, which avoided the potential autoimmune problems in future application. Above all, the expression of TgTH, as a functional protein, was expressed in the tachyzoites and bradyzoites of *T. gondii* and was distributed widely in all isolates despite geographic variation [16,21]. Consequently, TgTH is qualified as an ideal candidate for vaccine development based on the above advantages.

We found that a band with the molecular weight of 68 kDa in the somatic extraction of *T. gondii* tachychites was recognized by the serum against recombined TgTH. The molecular weight of native TgTH was a little bit larger than that of the deduced protein, which was 62.40 kDa. The protein of TgTH might be post-translationally modified since which possessed phosphorylation and glycosylation sites according to analyses of sequences.

According to the results of the Western Blots, serum samples from mice subjected to the experimental infection of *T. gondii* recognized the recombined TgTH, indicating that the immune system was able to recognize TgTH, which subsequently induced the expression of antibodies.

Protein localization in parasites helps to understand the function of localized proteins, and TgTH is generally believed to be secreted to host cells for catalytic activity. In the current study, it was verified that TgTH was located mainly at the surface of tachyzoites, which was consistent with the localization of TgTH in bradyzoites [16]. In addition, we found that TgTH could catalyze the production of dopamine, and this catalytic activity was dose dependent. However, the catalytic activity TgTH extracted from PRU strain *T. gondii* was significantly higher than that of rTgTH and TgTH extracted from RH strain *T. gondii*, which needs further study.

Toxoplasmosis is a serious global public problem that is caused by *T. gondii*. In previous studies, many *T. gondii* antigens have been proved to possess immunoprotective effects against acute and chronic toxoplasmosis [8]. In recent studies, lots of work has been done about DNA vaccines against toxoplasmosis [28,29,30]. In vivo protection has been considered as one of the most critical standards to assess the value of a candidate vaccine [25,31,32]. In order to evaluate the immunoprotective effect of rTgTH against toxoplasmosis, immunized BALB/c mice were intraperitoneally and intragastrically challenged with 1 × 10^3^ tachyzoite cells of the highly virulent RH *T. gondii* and 10 cysts of the chronic PRU *T. gondii*, respectively [29,33,34,35,36]. The surviving assays revealed that the survival rate of mice was significantly increased in the rTgTH immune group and that rTgTH as vaccine could remarkably decrease the number and the size of cysts in the brain, indicating the capacity of which inducing specific immunoreaction against high virulent and chronic *T. gondii* infection in the BALB/c mice model. The Toxoplasma strains were with different levels of virulence. In this study, we estimated the immunoprotective effect of rTgTH by challenging the RH and PRU *T. gondii*. Whether or not rTgTH can stimulate immune protection against other strains of *T. gondii* needs to be studied further.

Cytokines exert an essential role in activating Th cells [37]. Known as an inflammatory factor, IFN-γ resists the infection of pathogens and activates Th1 cells [38]. IL-2 promotes the differentiation of T cells into effector T cells and into memory T cells when the initial T cell is also stimulated by an antigen, thus helping the body fight off infections [39]. At the same time, IFN-γ is commonly taken as the marker that indicates the immune reaction of Th1 cells, which express large quantity of IL-2. However, in this research, TgTH could stimulate elevated expressions of IFN-γ, while the level of IL-2 was not improved. Whether TgTH as a vaccine could induce Th1 immune response need further research.

IL-4 promotes the division, differentiation, as well as the maturity of B cells, and the differentiation of the CD4^+^ T cell to Th2 cells, accompanied with antibody expressions, and it is considered a cytokine marker of Th2 cells [40]. Known as an important cytokine, IL-10 exerts an essential role in inflammation and regulating immunity. Moreover, the expressions of Th1 cytokines, MHC class II antigen and co-stimulatory molecules are down-regulated by the IL-10 of macrophages [41]. The antibody expression, proliferation, and survival of B cells are enhanced and the activity of NF-κB is blocked by IL-10 [42]. In addition, IL-10 participates in regulating the signaling pathway of JAK-STAT [43]. Xie et al. reported that the vaccine containing recombined α-actinin subunit of *T. vaginalis* as the antigen stimulated a significant increase in IL-10 levels [44]. In this study, the recombinant protein TgTH resulted in significantly increased IL-4 expression, although that of IL-10 was not elevated remarkably among mice subjected to TgTH vaccination.

Numerous immune regulatory functions have been reported for the IL-17 family of cytokines, presumably due to their induction of many immune signaling molecules [45,46]. The most notable role of IL-17 is its involvement in inducing and mediating proinflammatory responses [47]. IL-17 is commonly associated with allergic responses. IL-17 is expressed by Th17 cells, which is a kind of CD4^+^ cell [48]. In the current study, TgTH was capable of stimulating high IL-17 levels.

The expression of antibodies specific to pathogens, not only exerts an important role in regulating the immune reaction mediated by cells, but also suppresses parasites adhering to host cells through receptor molecules [31]. In addition, intracellular parasites are also coated and killed by macrophages which were recruited by these antibodies [32]. In the current study, in comparison to the mice receiving the vaccine composed of the control, the anti- *T. gondii* IgG expressions of those receiving the vaccine composed of rTgTH were increased. It was revealed by further analyses of IgG subgenera that the abundance of IgG1 was higher than that of IgG2a, suggesting that TgTH was capable of inducing an immune reaction mediated by Th2 cells and thus exerted an important effect on the immunity of hosts against *T. gondii*. However, the reason that TgTH could not stimulate IL-10 (Th2-type cytokine) quantity to be increased significantly needs further study.

## 5. Conclusions

The cloning of the TgTH gene and the expression of TgTH protein was completed. The enzyme activity test showed that TgTH could catalyze the substrate to produce dopamine in a dose-dependent manner, and the optimum catalytic temperature was 37 °C. Immunoblots revealed that TgTH had immunogenicity and could stimulate the production of specific antibodies. Immunofluorescence indicated that TgTH was located on the surface of the tachychites of *T. gondii*. It was indicated by the experiments of challenging animals that recombined TgTH protein prolongated survival duration after deadly challenging and reduced the number and size of brain cysts. Moreover, as a vaccine candidate antigen, TgTH stimulated the production of Th2 cell immunity. In brief, TgTH played an important role in increasing the neurotransmitter dopamine in the host brain and was capable of inducing partial immune protection against *T. gondii* acute and chronic infection.

## Figures and Tables

**Table 1:** The number and size of brain cysts in the mice after challenging with PRU *T. gondii*.

Groups	The Number of Brain Cysts (Mean ± SD)	Cysts Decrease Ratio (%)	The Size of Brain Cysts (px) (Mean ± SD)	Size Decrease Ratio (%)
Blank	2375 ± 884 ^a^	0.00 ^a^	518.80 ± 50.50 ^a^	0.00 ^a^
Freund adjuvant	2333 ± 520 ^a^	1.77 ^a^	492.30 ± 57.17 ^a^	5.11 ^a^
pET-32a	2313 ± 265 ^a^	2.61 ^a^	495.20 ± 61.77 ^a^	4.55 ^a^
TgTH	1275 ± 224 ^b^	46.32 ^b^	293.50 ± 46.45 ^b^	43.43 ^b^

Note: in each column, a significant difference between groups and ranks with different letters (*p* < 0.05), and no significant difference between groups and ranks with the same letter (*p* > 0.05).
